# Melanopsin, a Canonical Light Receptor, Mediates Thermal Activation of Clock Genes

**DOI:** 10.1038/s41598-017-13939-3

**Published:** 2017-10-25

**Authors:** Maria Nathália Moraes, Leonardo Vinícius Monteiro de Assis, Keila Karoline Magalhães-Marques, Maristela Oliveira Poletini, Leonardo Henrique Ribeiro Graciani de Lima, Ana Maria de Lauro Castrucci

**Affiliations:** 1 Department of Physiology, Institute of Biosciences, University of São Paulo, São Paulo, Brazil; 2 Department of Physiology and Biophysics, Institute of Biological Sciences, Federal University of Minas Gerais, Minas Gerais, Brazil; 3 Department of Biology, University of Virginia, Charlottesville, VA USA

## Abstract

Melanopsin (OPN4) is a photo-pigment found in a small subset of intrinsically photosensitive ganglion cells (ipRGCs) of the mammalian retina. These cells play a role in synchronizing the central circadian pacemaker to the astronomical day by conveying information about ambient light to the hypothalamic suprachiasmatic nucleus, the site of the master clock. We evaluated the effect of a heat stimulus (39.5 °C) on clock gene (*Per1 and Bmal1*) expression in cultured murine Melan-a melanocytes synchronized by medium changes, and in B16-F10 melanoma cells, in the presence of the selective OPN4 antagonist AA92593, or after OPN4 knockdown by small interfering RNA (siRNA). In addition, we evaluated the effects of heat shock on the localization of melanopsin by immunocytochemistry. In both cell lines melanopsin was found in a region capping the nucleus and heat shock did not affect its location. The heat-induced increase of *Per1* expression was inhibited when melanopsin was pharmacologically blocked by AA92593 as well as when its protein expression was suppressed by siRNA in both Melan-a and B16-F10 cells. These data strongly suggest that melanopsin is required for thermo-reception, acting as a thermo-opsin that ultimately feeds the local circadian clock in mouse melanocytes and melanoma cells.

## Introduction

The canonical role of melanopsin (OPN4) is to act as a photo-pigment in the mammalian intrinsically photosensitive retinal ganglion cells (ipRGCs)^1^. These cells play a role in synchronizing the central circadian pacemaker^2^ to the astronomical day by conveying information about ambient light to the hypothalamic suprachiasmatic nucleus (SCN), the site of the master clock^3^. Another function of melanopsin that was recently described is its participation in early visual system formation^4^. Upon photo-activation of mammalian ipRGCs, OPN4 triggers a signaling cascade leading to phospholipase C activation and subsequent opening of transient potential receptor channels, TRPC6/7, which ultimately leads to membrane depolarization^5^.

ipRGC axons release glutamate at the SCN neurons, increasing *Per* transcripts which reset the clock gene machinery. The biological mechanism of keeping track of time takes place through positive and negative interlaced feedback loops (reviewed in^6^). In summary, CLOCK and BMAL1 form a heterodimer that activates *Per* and *Cry* genes. PER and CRY proteins dimerize and after phosphorylation by casein kinases, are targeted to the nucleus, inhibiting the action of CLOCK/BMAL1. Once PER/CRY heterodimers are degraded, their inhibitory effect is reduced, and then CLOCK/BMAL1 is freed to start a new cycle of transcription. The core of clock gene machinery, described above, is stabilized by Rev-Erbα/β and RORα/β, whose transcripts are induced by CLOCK/BMAL1; Rev-Erbα/β inhibits while RORα/β activates *Bmal1* ^6^.

A local temporal controlling machinery has been found in almost every organ tested, comprising a multi-oscillatory system. These peripheral clocks are under the SCN control, which ensures that the whole organism is orchestrated in a single timing zone, allowing a harmonic working relationship among organs and systems^6–8^.

Interestingly, rhodopsin (OPN2), classically associated with image formation in arthropods and vertebrates^9^, has been demonstrated to participate in *Drosophila* temperature sensing. *Drosophila* larvae lacking rhodopsin lose the ability of thermo-discrimination^10–12^, which can be rescued by the targeted expression of mouse melanopsin^10^. In addition, melanopsin has been recently reported in murine blood vessels^13^, in which it mediates blue-light dependent photo-relaxation. Since the vascular physiology is under circadian control^14^, one may suggest that melanopsin could act as sensor that ultimately feeds the local temporal controlling system. Following this line, our group has shown that melanopsin and rhodopsin are expressed in murine melanocytes and melanoma cells where they may participate in a photo-sensitive system^15^. Based on these findings, we questioned whether an opsin could also function as a thermo-sensor in mammalian cells, conveying temperature information to the clock gene machinery of cutaneous melanocytes, cells known to be exposed to cycles of environmental light and temperature^16^.

## Results and Discussion

**Figure 1:**
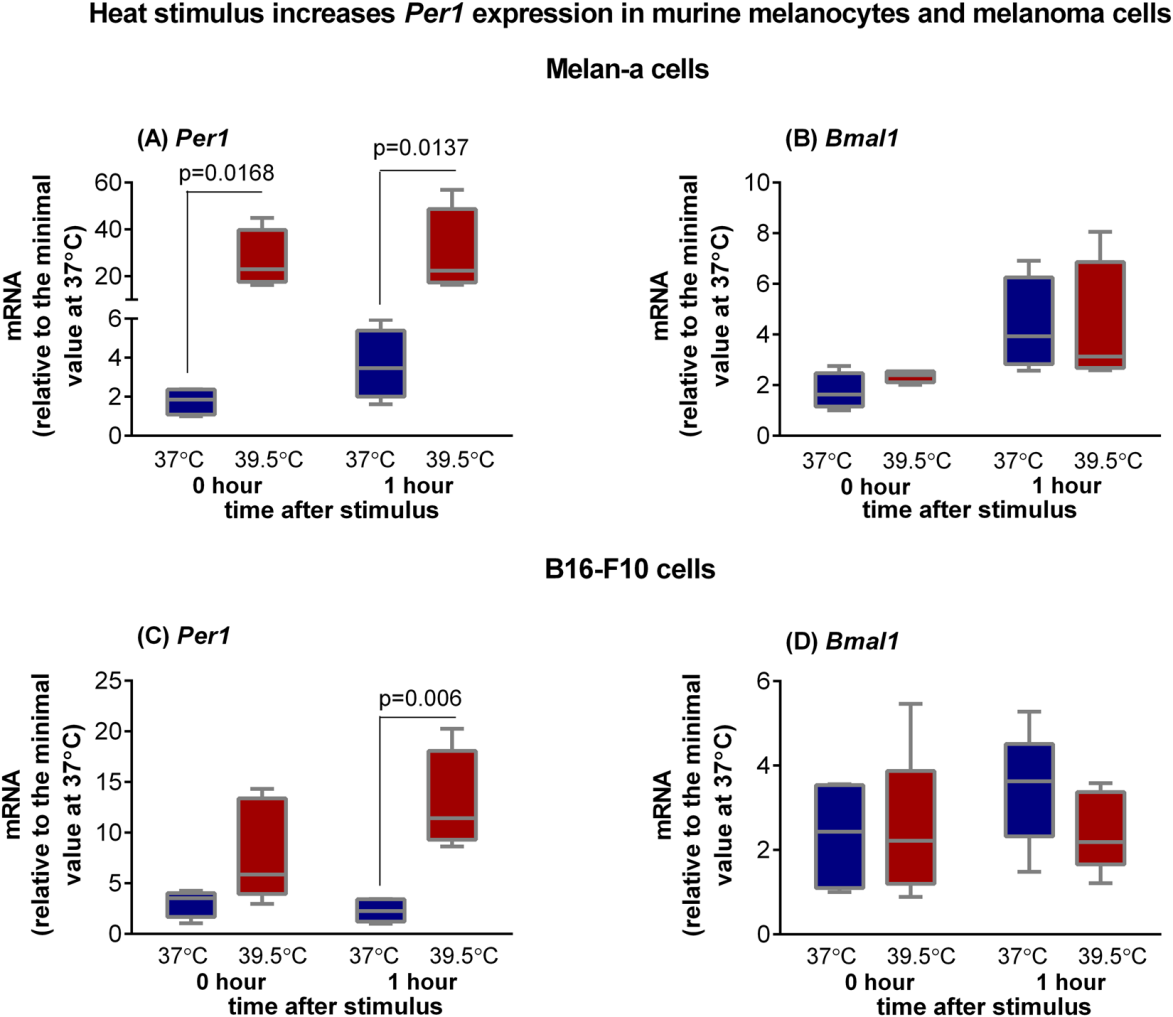
Expression of *Per1* (**A** and **C**) and *Bmal1* (**B** and **D**) in murine Melan-a melanocytes and B16-F10 melanoma cells after heat stimulus (39.5 °C). Melan-a or B16-F10 cells were kept for 3 days in constant dark and temperature (37 °C). In the beginning of the 4^th^ day, Melan-a cells were synchronized by two medium changes and after further 24 hours they were heat-stimulated (39.5 °C) during 1 h. B16-F10 cells were heat stimulated in the beginning of the 4^th^ day. Total RNA was extracted immediately and 1 h after the end of the stimulus for Melan-a and B16-F10 cells. Boxplots show the median, quartiles, maximum, and minimum expression values of each gene transcript normalized by 18S ribosomal RNA (for Melan-a cells) and *Rpl37a* (for B16-F10 cells), and expressed relative to the minimal value at 37 °C (N = 4–6). Statistical analysis was performed by Two-way ANOVA followed by Bonferroni post-test.

We evaluated the effect of a heat stimulus (39.5 °C) on clock gene expression in cultured Melan-a melanocytes and B16-F10 melanoma cells. Cells were maintained for three days under constant darkness and temperature (37 °C), a situation in which each cell displays its own rhythm of clock gene expression, usually leading to undetectable rhythm of the cell culture^17^. Melan-a cells exposed to 1 h heat pulse (39.5 °C) showed no difference from control cells in *Per1*, and *Bmal1* expression 0, 1 and 2 h after the stimulus (Supplemental Fig. 1). Because no effect was found in non-synchronized melanocytes, our next step was to repeat the same assay in cells synchronized by two medium changes^18,19^. In fact, 24 h after cell synchronization, heat shock led to increased *Per1* (0 and 1 h after the stimulus, Fig. 1A) but not *Bmal1* (Fig. 1B) expression. On the other hand, heat shock induced *Per1* increase in B16-F10 cells in constant dark condition 1 h after the end of the stimulus (Fig. 1C). Again, *Bmal1* was irresponsive to heat shock (Fig. 1D).

In agreement with these findings a 15-min white light pulse (WLP) applied to desynchronized Melan-a cells did not alter clock gene expression in comparison to cells kept in constant dark (DD) condition. Interestingly, in B16-F10 cells the expression of *Per1*, *Per2*, and *Bmal1* was upregulated in response to WLP^15^. Although the effects of heat shock or light pulse were not observed in desynchronized Melan-a cells, we cannot rule out that this stimulus may have affected the clock machinery of single cells, which would remain uncoupled to each other, and therefore, the overall oscillation would be unnoticed in the cell population. Another point of view is related to morpho-physiological differences between normal and malignant melanocytes, the latter showing denser dendritic projections among cells, a feature that probably allows more efficient cellular coupling^15^.

Temperature cycles have been shown to alter rhythmic parameters of clock genes in peripheral tissues^20^, and are a strong *zeitgeber* in normal murine keratinocytes^21^ and fibroblasts^22^. To our knowledge, this is the first report that a short heat pulse affects clock gene machinery in murine melanocytes and melanoma cells. In fact, in non-mammalian vertebrates, we have already shown that heat shock increases the expression of clock genes in the photosensitive teleost ZEM-2S cell line, but only when cells were synchronized by light/dark (LD) cycles^23^.

**Figure 2:**
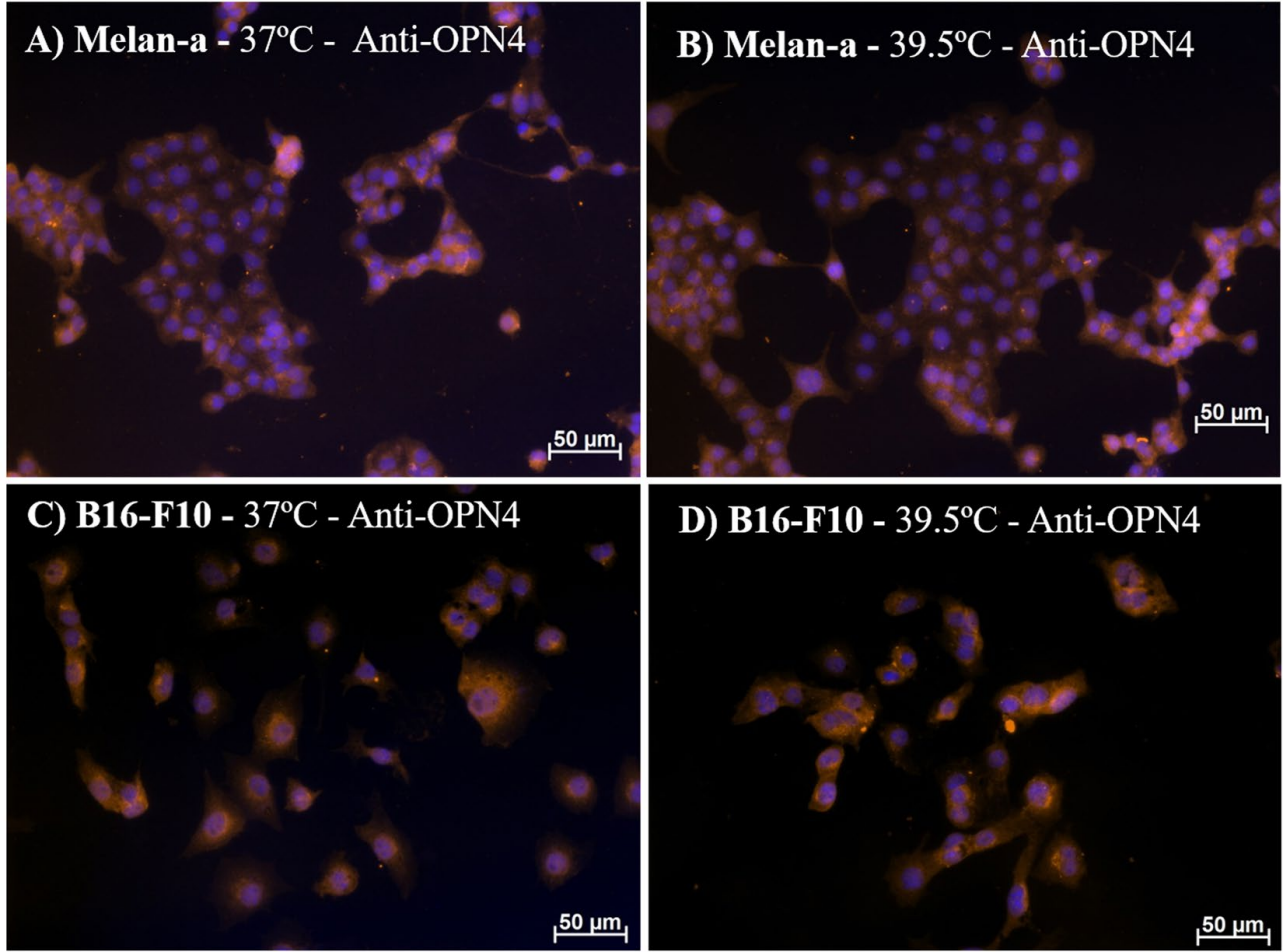
Representative fields of melanopsin (OPN4) immunostaining in Melan-a (**A**,**B**) and B16-F10 (**C**,**D**) cells. Cells were kept in DD for 3 days and at the beginning of the 4^th^ day, cells were divided into 2 groups: (1) Control group kept in constant dark and temperature (37 °C); (2) group in constant dark and exposed to 1 h heat stimulus (39.5 °C). Twenty-four hours later the medium was removed and the cells were fixed with 4% paraformaldehyde. DAPI stained nuclei in blue and OPN4 immunopositivity (1:500 antiserum), revealed with a Cy3-labeled secondary antibody, in orange. Photomicrographies were taken with Axiocam MRm camera (Zeiss) and pseudocolored with Axiovision software (Zeiss). Scale bar 50 μm (200x magnification).

Previous studies using Melan-a and B16-F10 cells^15^ and human and mouse spermatozoa^24^ showed immunolabeling of melanopsin in regions capping the nucleus. We have shown that a 15-min WLP promoted OPN4 translocation from nucleus region to the cytoplasm and cell membrane in B16-F10 cells 24 h after the stimulus^15^. Based on these findings, we investigated whether heat shock was also capable of inducing melanopsin translocation in Melan-a and B16-F10 cells. Our results demonstrate that the cytoplasm and the nucleus capping location of OPN4 in both cell lines was not altered 24 h after the heat shock of 39.5 °C (Fig. 2A–D), suggesting that OPN4 is not required to be inserted into the membrane to detect heat stimulus. In another line of thought, one may consider that the basal level of membrane-bound OPN4 may be enough to detect heat and trigger heat-induced responses in murine melanocytes and melanoma cells.

**Figure 3:**
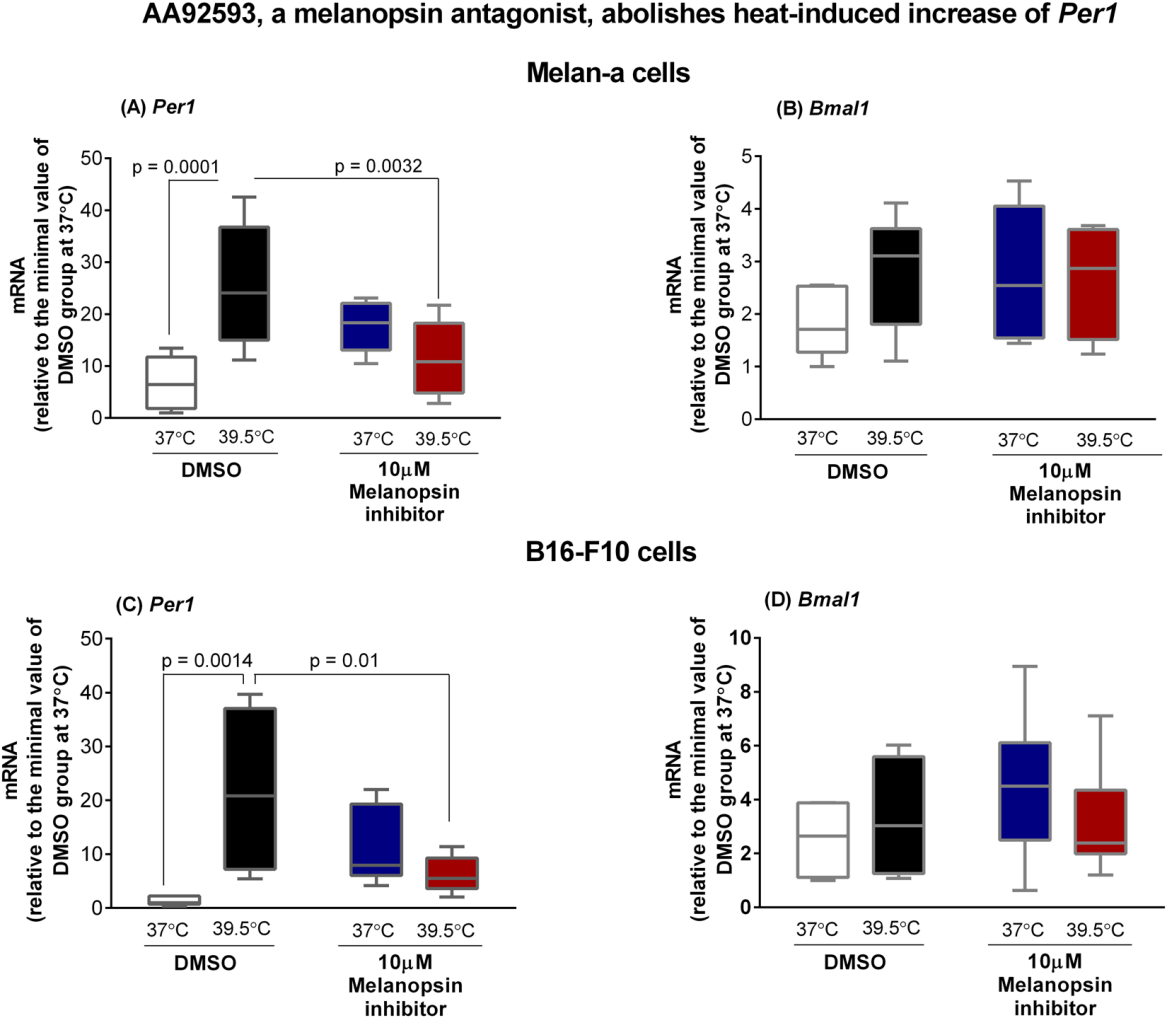
Expression of *Per1* (**A** and **C**) and *Bmal1* (**B** and **D**) in murine Melan-a melanocytes and B16-F10 melanoma cells after heat stimulus (39.5 °C) in the presence of AA92593. Melan-a or B16-F10 cells were kept for 3 days in constant dark and temperature (37 °C). In the beginning of the 4^th^ day, Melan-a cells were synchronized by two medium changes, and after further 24 hours cells were heat-stimulated. For B16-F10 cells, the heat shock (39.5 °C) was applied at the beginning of the 4^th^ day. In both scenarios, cells were divided into four groups: (1) control group at 37 °C in the presence of DMSO (0.1%); (2) heat-stimulated (39.5 °C) group in the presence of DMSO (0.1%); (3) group kept at 37 °C in the presence of AA92593 (10 µM), a selective OPN4 antagonist; (4) heat-stimulated (39.5 °C) group in the presence of AA92593 (10 µM). Total RNA was extracted immediately and 1 h after the end of the stimulus for Melan-a and B16-F10 cells, respectively. Boxplots show the median, quartiles, maximum, and minimum expression values of each gene transcript normalized by *Rpl 37a* and expressed relative to the minimal value of DMSO group at 37 °C (N = 5–9). Statistical analysis was performed by One-way ANOVA followed by Tukey’s test.

We then pharmacologically inhibited melanopsin with the antagonist AA92593, shown to be specific because it competes with retinaldehyde for the melanopsin retinal binding site which is very distinct from other opsins. Its administration to mice *in vivo* specifically and reversibly modified melanopsin-dependent light responses including the pupillary light reflex and light aversion^25^. Our data show that the increase of *Per1* level induced by heat in Melan-a and B16-F10 cells was significantly reduced in the presence of the melanopsin antagonist (Fig. 3A,C) whereas *Bmal1* expression was not affected (Fig. 3B,D). Surprisingly, in Melan-a cells the group incubated with the antagonist and kept at 37 °C showed a statistically significant increase of *Per1* transcript when compared to DMSO-treated control group (p = 0.0478, Fig. 3A); a phenomenon that showed no statistical significance (p = 0.08) in B16-F10 cells. The apparent intrinsic effect of AA92593 on control cells kept at 37 °C could be due to the following reasons: I) melanopsin acts as a thermosensor, and when inhibited, the cell would lose its ability to sense temperature, and the response would resemble the heat-evoked behavior; II) another possibility lies on a partial agonistic activity of AA92593 which, although not reported by Jones and co-workers^25^, could explain the response found in the control group treated with AA92593; III) since AA92593 is a competitive melanopsin antagonist, its presence in the retinal-binding pocket of melanopsin leads to the displacement of retinal, which could trigger a downstream signaling that would ultimately result in *Per1* increased expression.

**Figure 4:**
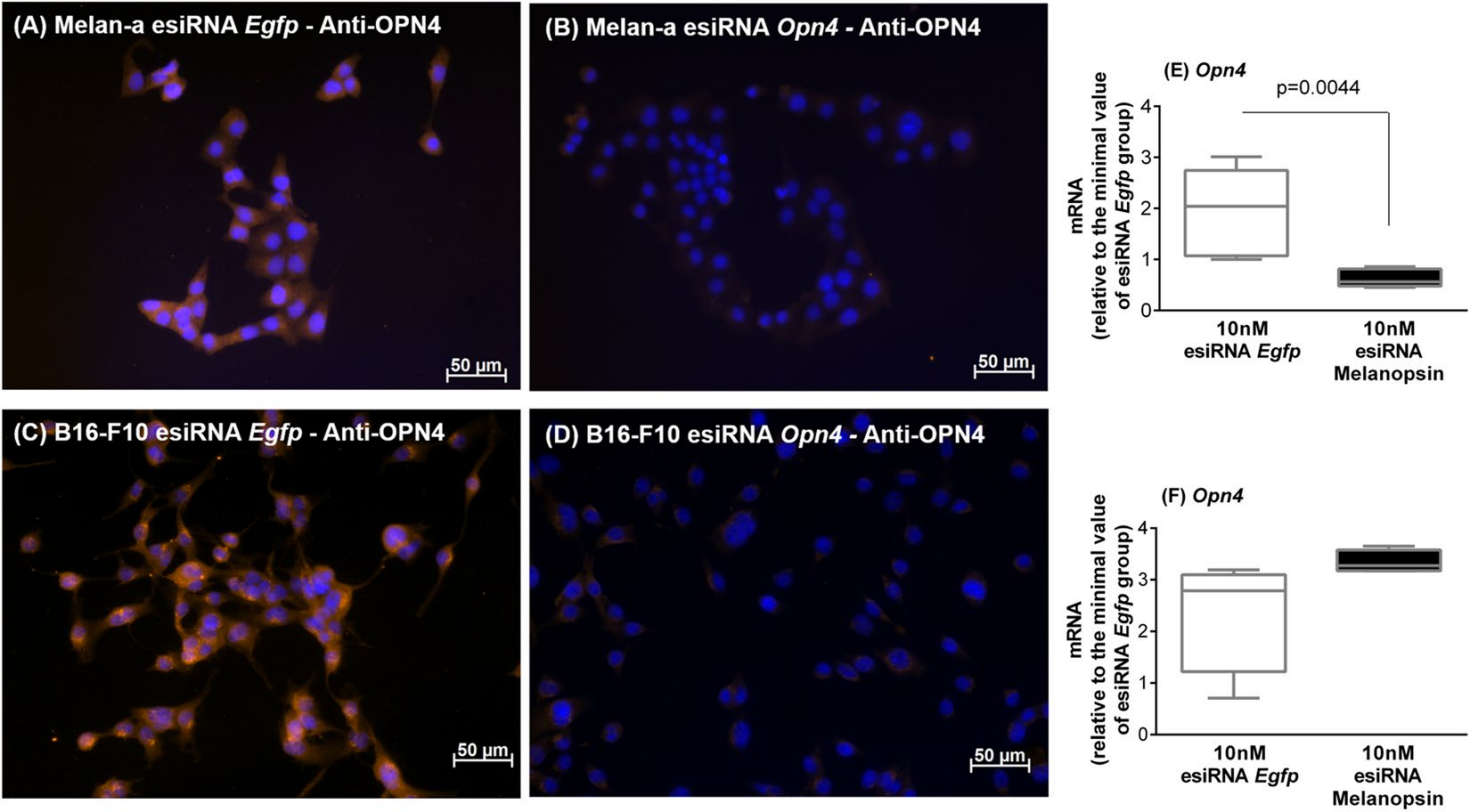
Representative fields of melanopsin (OPN4) immunostaining in Melan-a (**A**,**B**) and B16-F10 (**C**,**D**) cells. Cells were kept in DD for 24 hours, on the 2^nd^ day, were transfected with esiRNA against melanopsin or EGFP (both at 10 nM), and 48 h after transfection the cells were immunostained for melanopsin (OPN4). (**A** and **C**) esiRNA against mRNA of EGFP transfected cells (control group) and (**B** and **D**) esiRNA against mRNA of OPN4 transfected cells. Photo-micrographies were obtained with 200 x magnification in an inverted fluorescence microscope Axiovert 40CFL (Zeiss, Oberkochen, Germany) with a mercury lamp of 50 W, and DAPI (excitation 358 and emission 463 nm) and Cy3 (excitation 549 and emission 562 nm) filters. **Melanopsin gene and protein knockdown by endoribonuclease-prepared siRNAs (esiRNA)**. Gene expression of *Opn4* (melanopsin encoding gene) in esiRNA against mRNA of EGFP (control group) or of melanopsin transfected cells. Melan-a (**E**) or B16-F10 (**F**) cells were kept during three days in constant dark and temperature (37 °C). At the beginning of the 4^th^ day, Melan-a cells were synchronized by two medium changes, and after further 24 hours they were transfected with esiRNA. B16-F10 cells were transfected at the beginning of the 4^th^ day. In both cases, gene expression was evaluated 48 h after transfection with esiRNA. Boxplots show the median, quartiles, maximum, and minimum expression values of each gene transcript normalized by *Rpl 37a* and expressed relative to the minimal value of the esiRNA EGFP group kept at 37 °C (N = 5–6). Statistical analysis was performed by Student’s t test.

**Figure 5:**
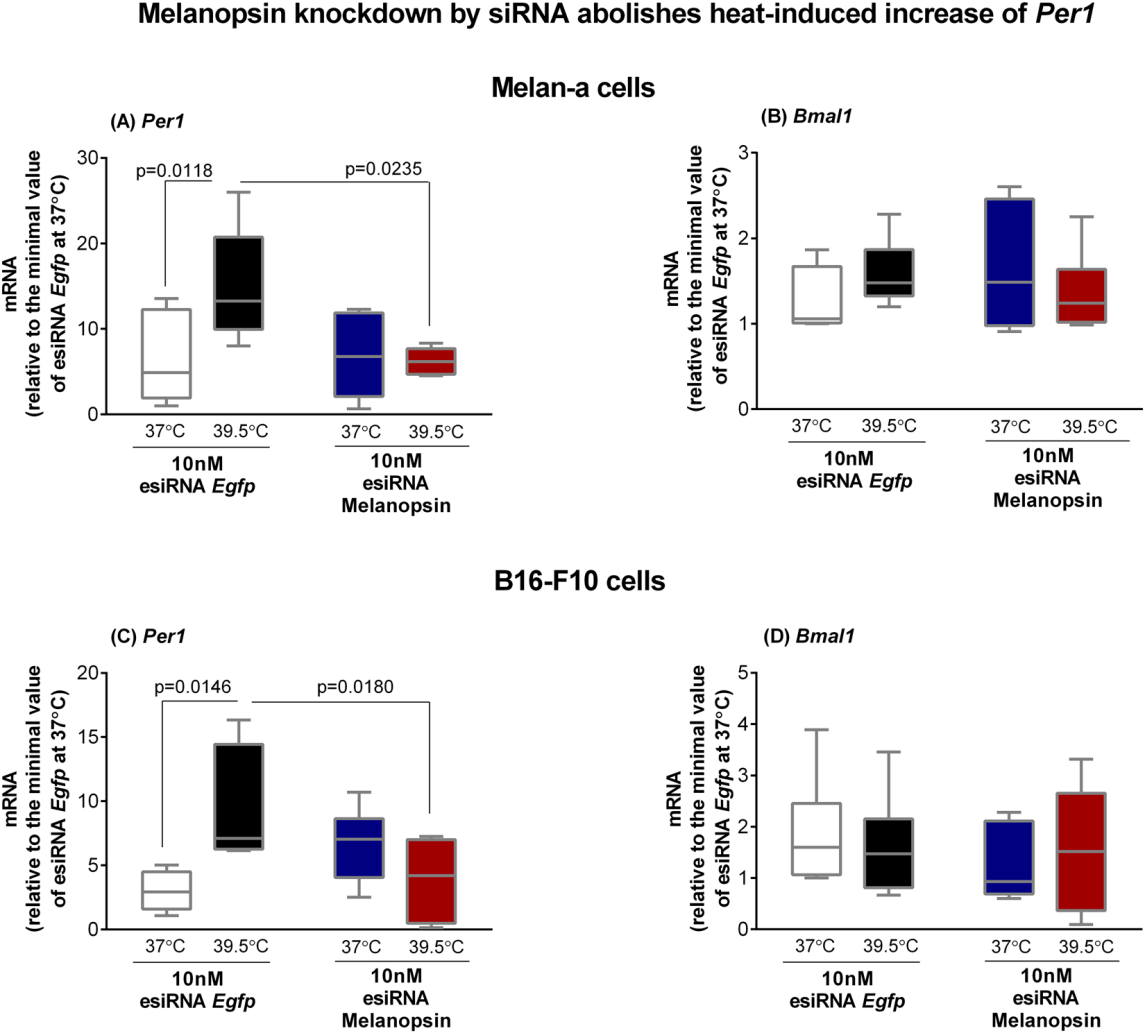
Expression of *Per1* (**A** and **C**) and *Bmal1* (**B** and **D**) in esiRNA transfected murine Melan-a melanocytes and B16-F10 melanoma cells after heat stimulus (39.5 °C). Melan-a or B16-F10 cells were kept during three days in constant dark and temperature (37 °C). At the beginning of the 4^th^ day, Melan-a cells were synchronized by two medium changes, and after further 24 h cells were transfected with esiRNA against melanopsin or EGFP (both at 10 nM) using Lipofectamine 3000 transfection kit. B16-F10 cells were transfected with esiRNA, as described above, at the beginning of the 4^th^ day. In both experimental scenarios, 48 hours after transfection, cells were divided into four groups: (1) control group at 37 °C in the presence of esiRNA EGFP (10 nM); (2) heat-stimulated (39.5 °C) group in the presence of esiRNA EGFP (10 nM); (3) group at 37 °C in the presence of esiRNA melanopsin (10 nM); (4) heat-stimulated (39.5 °C) group in the presence of esiRNA melanopsin (10 nM). Boxplots show the median, quartiles, maximum, and minimum expression values of each gene transcript normalized by *Rpl 37a* and expressed relative to the minimal value of the esiRNA EGFP group at 37 °C (N = 5–11). Total RNA was extracted immediately and 1 h after the end of the stimulus for Melan-a and B16-F10 cells, respectively. Statistical analysis was performed by One-way ANOVA followed by Tukey’s test.

Next, we performed gene knockdown assays using the endoribonuclease-prepared siRNAs (esiRNA), which consist of a heterogeneous mixture of siRNAs against murine melanopsin mRNA. These siRNAs selectively suppress gene expression with low off-target effects^26^. In fact, we show a significant reduction of *Opn4* transcription as well as melanopsin protein level 48 h after transfection in Melan-a cells (Fig. 4A,B,E). In B16-F10 cells, although the mRNA levels of melanopsin did not decrease 48 h after transfection (Fig. 4F), a fact that could be due to a faster mRNA turnover, OPN4 protein level was drastically reduced (Fig. 4C,D). Then, our next step was to heat shock Melan-a and B16-F10 cells with reduced melanopsin expression, and the results demonstrated that the heat-induced increase of *Per1* was significantly reduced in both cell lines (Fig. 5A,C) while no effect on *Bmal1* expression was found (Fig. 5B,D). We have demonstrated that an opsin in a skin cell type can be activated not only by photons^15^ but also by thermal energy. In fact, thermo-isomerization of rhodopsin and cone opsins has been shown to occur and it requires about half the energy necessary to photo-activate the photo-pigment^27^. Therefore, taken together our results clearly demonstrate– for the first time – the involvement of melanopsin in thermo-responses of mammalian cells.

According to Colin Pittendrigh *Escape from Light* theory^28^, higher temperatures are found during the photo-phase of the day and, therefore, temperature and light are environmental entities exerting simultaneous selective pressures on the organisms. It is not surprising, therefore, that light and temperature may be perceived by the same conserved proteins, having in mind that photo- and thermo-sensitive systems probably co-evolved during evolution. Considering that the skin is constantly exposed to both physical stimuli^16^, it is relevant to better understand how the skin perceives heat and light. In fact, our data add another layer of complexity for this system: an opsin, which is classically a light sensor, also acts as a thermo-sensor that ultimately feeds the local circadian clock. Interestingly, the opsin-mediated clock gene activation is conserved in malignant melanocytes, which warrants further investigation whether this event was pro- or anti-tumorigenic. Within this line, it has been recently shown that clock gene machinery of melanoma cells is suppressed^15,29^, but clock gene activation by dexamethasone, forskolin, or heat shock results in reduced melanoma proliferation *in vitro* and *in vivo* without leading to cell death^29^. Taken altogether, our data bring convincing evidence of a new role for a canonical mammalian light sensor, which challenges the current paradigm that mammalian opsins exclusively function as photo-pigments.

## Material and Methods

### Cell Culture

Immortalized murine Melan-a melanocytes and B16-F10 malignant melanocytes were cultured in RPMI 1640 medium without phenol red (Atená, Campinas, SP, Brazil), supplemented with 14.3 mM NaHCO_3_, 15 mM HEPES, 10% fetal bovine serum (FBS) (Atená, Campinas, SP, Brazil), 1% antibiotic/antimycotic solution (10,000 U/mL penicillin, 10,000 μg/mL streptomycin, and 25 μg/mL amphotericin B, ThermoFisher, Waltham, MA, USA), and 100 nM of *all-trans* retinal (Sigma-Aldrich, St. Louis, MO, USA). Phorbol 12-myristate 13-acetate (TPA, Sigma-Aldrich, St. Louis, MO, USA) at 200 nM was added to Melan-a medium, since it is required to maintain cell viability in culture^30^. The pH was adjusted to 7.2, and the cells were kept at constant temperature (37 °C) with 5% CO_2_. Previous cell maintenance and experiment set up were carried out under ambient lighting.

### Experimental Design

In all experiments, Melan-a and B16-F10 cells were maintained in the medium described above but FBS was reduced to 2% (experimental medium). Cell manipulation during the experiments was carried out under red dim light (7 W Konex bulb and Safe-Light filter GBX-2, Kodak, Rochester, NY, USA). For all protocols described below, Melan-a and B16-F10 cells were kept in constant dark and temperature (37 °C) during 3 days. At the beginning of the 4^th^ day the medium of Melan-a cells was changed twice with a 2-hour interval, after which the cells were kept in DD during further 24 h. This procedure has been shown to synchronize clock genes in this cell line^31^.

### Effect of Heat Stimulus on Clock Gene Machinery of Melan-a and B16-F10 Cells

Melan-a or B16-F10 cells were seeded at the density of 10^6^ and 10^5^ cells respectively in 25 cm^2^ flasks. In the beginning of the 4^th^ or 5^th^ day (24 h after medium changes), B16-F10 and Melan-a cells were, respectively, heat-stimulated (39.5 °C) during 1 h while the control group remained at 37 °C. Total RNA was initially extracted immediately, 1 and 2 h after the end of the heat stimulus, and the time points showing maximal response of clock gene expression were adopted for subsequent assays.

### Effect of Heat Stimulus on OPN4 Localization in Melan-a and B16-F10 Cells

Immunocytochemistry assays were performed as previously described^15^. A peptide comprised by the 15 N-terminal amino acid sequence of mouse melanopsin (Genbank accession NP_038915) with an appended C-terminal cysteine (MDSPSGPRVLSSLTQC) (Uniformed Services University of the Health Biomedical Instrumentation Center, Bethesda, MD, USA) was conjugated to keyhole limpet hemocyanin and used to immunize rabbits (Covance Labs, Denver, PA, USA). The antisera were used with no further purification^1^. Previous studies showed that increasing the concentration of the antigenic peptide led to the loss of immunoreactivity by pre-absorption in a dose-dependent manner, and that retinas of OPN4 knockout mice showed lack of immunoreactivity^32^.

Melan-a or B16-F10 cells were seeded (10^4^/well) into 8-chamber slides in the experimental medium as described above. The cells were kept in DD at 37 °C for 3 days and at the beginning of the 4^th^ day they were divided into 2 groups: the control remained in DD at 37 °C while the experimental group was exposed to 1 h of heat stimulus (39.5 °C). Twenty-four hours later the medium was removed and the cells were fixed in 4% paraformaldehyde as described below.

To verify the effectiveness of the *Opn4* silencing Melan-a or B16-F10 cells were seeded (10^4^/well) into 8-chamber slides and kept in DD for 24 hours. On the 2^nd^ day, the cells were transfected with esiRNA, and immunostaining of OPN4 was performed 48 h after transfection. The cells were incubated in the primary antibody anti-melanopsin (1:500, Covance Laboratories, Denver, PA, USA) overnight at 4 °C. A Cy3-labeled anti-rabbit secondary antibody (1:500, Jackson Immunolab, West Grove, PA, USA) was applied for 1 h at room temperature. Photo-micrographies were obtained with 200 x magnification in an inverted fluorescence microscope Axiovert 40CFL (Zeiss, Oberkochen, Germany) with a mercury lamp of 50 W, and DAPI (excitation 358 and emission 463 nm) and Cy3 (excitation 549 and emission 562 nm) filters.

### Pharmacological Inhibition of Melanopsin

We used the selective competitive antagonist of melanopsin, AA92593 (Sigma-Aldrich, St. Louis, MO, USA), at 10 µM, based on a previous study^25^. Melan-a and B16-F10 cells were seeded at the density of 10^6^ and 10^5^ cells respectively in 25 cm^2^ flasks, and after the procedure described in the Experimental Design section, they were divided into four groups: (1) control group at 37 °C in the presence of DMSO (0.1%); (2) heat-stimulated group (39.5 °C) in the presence of DMSO (0.1%); (3) group kept at 37 °C in the presence of the OPN4 antagonist AA92593 (10 µM); (4) heat-stimulated group (39.5 °C) in the presence of AA92593 (10 µM). Total RNA of Melan-a cells was extracted immediately and of B16-F10 cells 1 h after the end of the heat stimulus.

### Melanopsin Knockdown by Endoribonuclease Small Interfering RNA (esiRNA)

We used esiRNA as gene silencing tool (Sigma-Aldrich, St. Louis, MO, USA) that targets mouse *Opn4* variants 1 and 2 (access numbers NM_001128599.1 and NM_013887.2). The esiRNA results from the cleavage of long double-stranded RNA (dsRNA). This process generates a heterogeneous mixture of siRNAs, all of which target the same mRNA of interest. This methodology provides highly selective gene suppression with lower off-target effects than single or pooled siRNAs^26^. As a control for our experiments, we used an esiRNA that targets the mRNA of Enhanced Green Fluorescent Protein (EGFP), which can be used as a negative control in systems that lack this protein.

Melan-a or B16-F10 cells were seeded (10^5^ and 5 × 10^4^/well respectively) in a 12-well plate, and after the procedure described in the Experimental Design section they were transfected with esiRNA against melanopsin or EGFP (both at 10 nM) using Lipofectamine 3000 transfection kit (ThermoFisher, Waltham, MA, USA) according to the manufacturer’s instructions.

To verify the functional role of melanopsin in perceiving heat, Melan-a or B16-F10 cells were divided into four groups 48 hours after transfection: (1) control group transfected with esiRNA EGFP (10 nM) at 37 °C; (2) heat-stimulated (39.5 °C) group transfected with esiRNA EGFP (10 nM); (3) group transfected with esiRNA melanopsin (10 nM) at 37 °C; (4) heat-stimulated (39.5 °C) group transfected with esiRNA melanopsin (10 nM). Total RNA was extracted immediately after the end of heat stimulus and gene expression of *Opn4* (melanopsin encoding gene) was assessed by qPCR as previously described^15^ in Melan-a and B16-F10 cells transfected with esiRNA against EGFP (control group) or against melanopsin mRNA. Lipofectamine 3000 displayed no effect *per se* on clock gene expression (data not shown).

### RNA Extraction, Purification, and cDNA Synthesis

RNA was extracted with trizol (Ambion, Carlsbad, CA, USA), and purified (Direct-zol™ Zymo Research, Irvine, CA, USA) according to the manufacturers’ instructions. RNA concentration and quality (OD260/OD280) were determined in a NanoDrop spectrophotometer (NanoDrop, Wilmington, DE, USA), and 1 µg of total RNA was reverse transcribed to cDNA using random hexamer primers and *Superscript III*, following the manufacturer’s instruction (ThermoFisher, Waltham, MA, USA).

### Quantitative PCR

Quantitative PCR reactions were performed in an iQ5 thermocycler (Bio-Rad Laboratories, Hercules, CA, USA) with the products of reverse transcription using primers spanning introns, designed, and synthesized by IDT (Coralville, IA, USA), and based on sequences obtained from GenBank (http://www.ncbi.nlm.nih.gov/genbank). The access number of each gene, the respective primer sequences, and concentrations are shown in Table 1. The qPCR reactions were performed using two different protocols: multiplex for simultaneous analysis of multiple genes (TaqMan®) and SYBR® GreenER™. The TaqMan® solutions contained *Per1* and *Bmal1* respective primers and fluorescent probes (Table 1), and iQ Multiplex Powermix (Bio-Rad Laboratories, Hercules, CA, USA) or Kapa Probe Fast qPCR Mix 2X (Kapa Biosystems, Wilmington, MA, USA). Each experimental cDNA was run in triplicates (1 µl of cDNA per reaction) in 96 well plates. The assays were performed under the following conditions: 7 min at 95 °C, followed by 45 cycles of 30 s at 95 °C and 30 s at 55 °C.

**Table 1:** Sequences and Final Concentrations of Primers and Probes. Access Numbers in Between Parentheses.

**Templates**	**Primers and probes**	**Final Concentration**
*Per1* (NM_0011065.3)	Forward: 5′-AGCAGGTTCAGGCTAACCAGGAAT-3′	300 nM
	Reverse: 5′-AGGTGTCCTGGTTTCGAAGTGTGT-3′	300 nM
	Probe:5′-/6FAM/AGCCTTGTGCCATGGACATGTCTACT/BHQ_1/-3′	200 nM
*Bmal1* (NM_001243048)	Forward: 5′-AGCTTCTGCACAATCCACAGCAC-3′	300 nM
	Reverse: 5′-TGTCTGGCTCATTGTCTTCGTCCA-3′	300 nM
	Probe:5′-/5HEX/-AAAGCTGGCCACCCACGAAGATGGG/BHQ_1–3′	200 nM
*Opn4* (NM_001128599.1)	Forward: 5′-ACATCTTCATCTTCAGGGCCA-3′	300 nM
	Reverse: 5′-ACTCACCGCAGCCCTCAC-3′	300 nM
*Rpl37a* (NM_009084.4)	Forward: GCATGAAAACAGTGGCCGGT	300 nM
	Reverse: CAGGGTCACACAGTATGTCTCAAAA	300 nM
*18S RNA*	Forward: 5′-CGGCTACCACATCCAAGGAA-3′	50 nM
	Reverse: 5′-GCTGGAATTACCGCGGCT-3′	50 nM

The solutions for *Opn4*, *Rpl 37a* or 18S RNA contained the respective primers (Table 1) and Kapa SYBR® Fast qPCR Master Mix 2X (Kapa Biosystems, Wilmington, MA, USA). Each experimental cDNA was run in duplicates (1 µl of cDNA per well) in 96 well plates. These assays were performed under the following conditions: 10 min at 95 °C, followed by 45 cycles of 15 s at 95 °C, 1 min at 60 °C, and 80 cycles of 10s at 55 °C, with a gradual increase of 0.5 °C. Ribosomal 18S RNA and *Rpl 37a* were used as reference genes in both TaqMan® and SYBR® GreenER™ methodologies since they did not vary with time under our experimental conditions.

### Statistical Analyses

To analyze qPCR data, we used the 2^−ΔΔCT^ method as previously described^33^. The temporal effect of heat stimulus on clock gene expression of Melan-a and B16-F10 cells was analyzed by Two-way ANOVA followed by Bonferroni post-test. For the pharmacological and gene knockdown assays, Student’s *t* test or One-way ANOVA followed by Tukey post-test were used according to the number of groups. Significance was set for p < 0.05. All analyses were carried out in GraphPad Prism Version 6.0 (La Jolla, CA, USA).

## Electronic supplementary material

Supplementary Figure 1
