# Supplementary material for: Melanopsin, a Canonical Light Receptor, Mediates Thermal Activation of Clock Genes
Source: Sci Rep. 2017 Oct 25;7:13977. doi: 10.1038/s41598-017-13939-3 (PMC5656685; doi:10.1038/s41598-017-13939-3)
Supplement: Supplementary file 1 — Supplementary Figure 1 [file 41598_2017_13939_MOESM1_ESM.pdf]

Melanopsin, a Canonical Light Receptor, Mediates Thermal Activation of Clock Genes

Maria Nathália Moraes[1*], Leonardo Vinícius Monteiro de Assis[1*], Keila Karoline Magalhães-Marques[1], Maristela Oliveira Poletini[2], Leonardo Henrique Ribeiro Graciani de Lima[1], Ana Maria de Lauro Castrucci[1,3]

[1] Department of Physiology, Institute of Biosciences, University of São Paulo, São Paulo, Brazil

[2] Department of Physiology and Biophysics, Institute of Biological Sciences, Federal University of Minas Gerais, Minas Gerais, Brazil.

[3] Department of Biology, University of Virginia, Charlottesville, VA, USA

*Moraes, M.N. and de Assis, L.V.M contributed equally to this paper.

[#]**Corresponding author**: Ana Maria de Lauro Castrucci

Departamento de Fisiologia

Instituto de Biociências

Universidade de São Paulo

R. do Matão, trav. 14, no. 101

São Paulo, 05508-900, Brazil

Telephone: 5511-3091-7523

Email: amdlcast@ib.usp.br

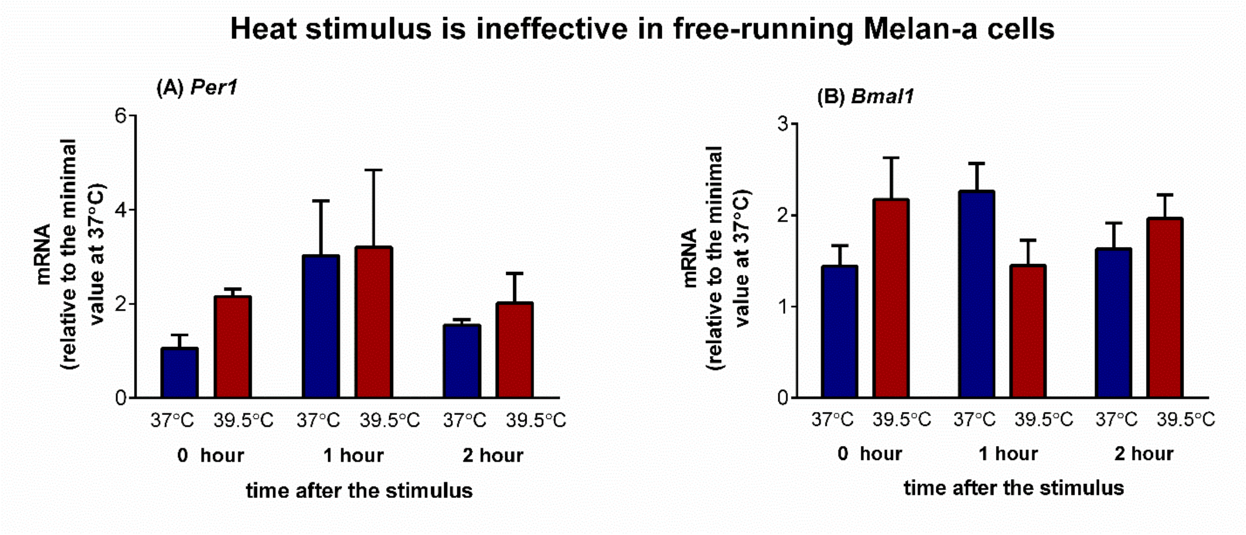

Supplementary Figure 1. Expression of *Per1* (A) and *Bmal1* (B) in non-synchronized murine Melan-a melanocytes after heat stimulus (39.5°C). Melan-a cells were seeded at the density of 10^6 in 25 cm^2 flasks, and kept for 3 days in constant dark and temperature (37°C). In the beginning of the 4th day, Melan-a cells were heat stimulated (39.5°C) during one h. Total RNA was extracted immediately, 1 and 2 h after the end of the stimulus. Values are shown as mean ± SEM of each gene transcript normalized by 18S ribosomal RNA and expressed relative to the minimal value at 37°C (N=3-6). Statistical analysis was performed by Two-way ANOVA followed by Bonferroni post-test.
